# Achieving Validated Thresholds for Clinically Meaningful Change on the Knee Injury and Osteoarthritis Outcome Score After Total Knee Arthroplasty: Findings From a University-based Orthopaedic Tertiary Care Safety Net Practice

**DOI:** 10.5435/JAAOSGlobal-D-19-00142

**Published:** 2019-11-04

**Authors:** Adam Haydel, Seth Guilbeau, Ryan Roubion, Claudia Leonardi, Amy Bronstone, Vinod Dasa

**Affiliations:** From the LSUHSC School of Medicine (Mr. Haydel and Mr. Guilbeau); the Department of Orthopaedics (Dr. Roubion and Dr. Dasa), LSUHSC School of Medicine; the LSUHSC School of Public Health (Dr. Leonardi), New Orleans, LA; and the BioMedEcon, Moss Beach, CA (Dr. Bronstone).

## Abstract

A lack of knowledge exists about which patient characteristics predict failure to meet validated thresholds for clinically meaningful change on the Knee Injury and Osteoarthritis Outcome Score (KOOS) after total knee arthroplasty (TKA).

**Methods:**

A retrospective chart review was performed on patients who underwent primary TKA by a single surgeon between January 2013 and June 2018. Variables included demographics (age, sex, race, and insurance type), comorbidities, body mass index, and preoperative KOOS subscale scores. Multivariate logistic regression was performed to identify characteristics associated with failing to meet or exceed the minimal clinically important difference (MCID) and substantial clinical benefit (SCB) on each KOOS subscale 6 months after TKA.

**Results:**

A total of 159 patients were included. At 6 months after TKA, approximately one-third of patients (21% to 32%) failed to meet or exceed the MCID and 27% to 39% failed to meet or exceed the SCB on all KOOS subscales. Better preoperative KOOS Symptoms, quality of life, and activities of daily living subscale scores were statistically significantly associated with failing to meet the MCID and SCB on each respective subscale. Demographics, comorbidities, and body mass index were not notable predictors of either outcome for any of the KOOS subscales.

**Discussion:**

About one-third of TKA patients in this single-site, single-surgeon sample failed to achieve a clinically meaningful outcome, and up to 4 in 10 patients had a less-than-ideal outcome 6 months after surgery. Surgeons should take care to set realistic expectations for patients with the least severe knee problems before TKA because this subgroup is especially at a high risk of failing to achieve a satisfactory outcome.

The economic burden of total knee arthroplasty (TKA) is substantial given its high frequency (about 600,000 TKAs are performed each year in the United States) and average cost of about $31,000.^1,2^ Although TKA is generally effective in relieving osteoarthritis-related symptoms,^3^ 10% to 30% of TKA patients report having persistent pain and functional limitations after surgery.^4,5^ Understanding the determinants of suboptimal TKA outcomes can help inform patient selection, preparation, and education (eg, preoperative rehabilitation and setting expectations) to increase the proportion of patients with clinically meaningful improvement after TKA. In addition, the ability to identify patients at risk for a poor outcome could help inform policies for risk adjustment of bundled payments, such as the Centers for Medicare and Medicaid Services' Comprehensive Care for Joint Replacement model. The Comprehensive Care for Joint Replacement model will incorporate patient-reported outcome (PRO) measures as a performance metric and hold hospitals and surgeons financially accountable for outcomes 1 year after TKA.^6,7^

Because PROs that evaluate TKA outcomes are typically continuous measures, and there have been no well-established thresholds for determining the amount of change over time that is clinically meaningful, such measures have limited utility in clinical decision making. The Knee Injury and Osteoarthritis Outcome Score (KOOS) is a commonly used PRO measure to assess change in knee pain, symptoms, function, and quality of life (QOL) after TKA. To help clinicians better understand how to interpret changes in the KOOS over time, Lyman et al^8^ validated thresholds for the minimal clinically important difference (MCID) and substantial clinical benefit (SCB) for each subscale of the KOOS using anchor-based methods. The MCID reflects the minimal change in a KOOS subscale that patients perceive as a benefit to their health; patients who meet or exceed the MCID can be considered as having had an adequate outcome. The SCB establishes a higher threshold for success and is based on the minimal change in each KOOS subscale associated with patients who consider their outcome “more improvement than I ever dreamed possible” or “great improvement”.^8^ Patients with KOOS subscale scores that meet or exceed the SCB can be considered as having an excellent outcome.

Various studies have sought to identify the most important preoperative factors that predict PROs 6 months to 5 years after TKA, including patient demographics, psychological and social factors (eg, anxiety, depression, social support, and expectations), and clinical factors (eg, body mass index [BMI], comorbidities, preoperative pain, and function).^9,10^ In multivariate analyses, preoperative pain and function are the most consistent independent predictors of TKA outcomes assessed ≥6 months after surgery; greater preoperative pain and worse preoperative function strongly predict less pain and better physical function at follow-up.^11,12,13,14,15^

Knowing that patients with worse pain and function tend to have better outcomes after TKA has little clinical utility, unless physicians can identify which subgroup of patients is at risk for an inadequate or less-than-ideal outcome using validated thresholds for the MCID and SCB, respectively. To the best of our knowledge, this is the first study to examine patient factors that predict the MCID and SCB, as established by Lyman et al, for the KOOS after TKA.

## Methods

### Study Design

A retrospective chart review was conducted on all patients who underwent primary unilateral TKA between January 1, 2013, and June 1, 2018, by a single orthopaedic surgeon at a university-based orthopaedic tertiary care safety net practice. Each patient completed the KOOS before surgery and 6 months after TKA. Patients who had Medicaid or other insurance, who reported a race other than white or black, and who lacked complete preoperative or 6-month postoperative KOOS data were excluded. The study was approved by an institutional review board.

All patients underwent navigated TKA using Zimmer Persona, Zimmer NexGen, or United U2 cemented implants.

### Measures

Data abstracted from patient charts included demographics (age, sex, race, and type of insurance), the Charlson Comorbidity Index (CCI), BMI, and KOOS subscale scores collected before surgery and 6 months after TKA.

The 42-item KOOS has five subscales (Pain, Symptoms, Function of Daily Living [activities of daily living (ADL)], Function in Sport and Recreation, and Knee-related QOL), with individual items assessed using a 5-point Likert scale in which 0 indicates no problems and 4 indicates extreme problems. The raw scores are converted to a 0 to 100 scale in which 0 represents extreme problems and 100 represents no problems.^16^ The KOOS Function in the Sport and Recreation subscale has negligible value for evaluating TKA outcomes in this setting, as most patients are not normally engaging in activities measured by this subscale (eg, running and jumping) and so was assessed but not analyzed as part of this study.

The main outcomes were failing to meet the MCID and SCB of each KOOS subscale, as defined by Lyman et al.^8^ The MCID and SCB, respectively, for each of the KOOS subscales were 7 and 21 (KOOS Symptoms), 18 and 22 (KOOS Pain), 16 and 15 (KOOS ADL), and 17 and 23 (KOOS QOL).^8^

Patients' preoperative KOOS subscale scores were grouped by quartiles, with those with the worst preoperative scores (first quartile) as the reference group. The change in each KOOS subscale score from baseline (before surgery) to 6-month follow-up was calculated for each patient.

### Statistical Analyses

Data were analyzed using SAS/STAT software version 9.4 (SAS Institute, Cary, NC). Descriptive statistics were calculated for demographic and baseline characteristics. Multivariate logistic regression analyses were performed to identify patient characteristics predicting an inadequate (MCID) and less-than-ideal (SCB) outcome on each KOOS subscale. The patient characteristics investigated as fixed effects were race (white and black), sex, insurance type (private, Medicare Advantage, and Medicare), age (<65 years, 65 to 74 years, and ≥75 years), BMI (<25 kg/m^2^, 25 to 29 kg/m^2^, 30 to 34 kg/m^2^, and ≥ 35 kg/m^2^), CCI (0 to 1, 2 to 3, and >3), and KOOS preoperative subscale scores by quartiles. Year of surgery was included in the model as a random effect to account for possible variability over time. A *P* value of <0.05 was considered statistically significant.

## Results

### Patient Characteristics

Among the 309 patient charts reviewed, 28 were excluded because of either not reporting their race or having a race other than white or black (n = 14), either not reporting insurance or having an insurance type other than private, Medicare Advantage, or Medicare (n = 12), or both (n = 2). The number of patients in the other race and other insurance subgroups was too low to compare with other subgroups, and so, patients in these subgroups were excluded from analyses. Of the remaining 281 patients, 159 patients completed a KOOS questionnaire both at baseline and 6-month follow-up and were included in the study. Table 1 shows the demographic and clinical characteristics of the study sample. Most patients were women (69.2%), with an average age of 67.6 years (range, 45 to 87 years) and mean BMI of 31.7 kg/m^2^ (range, 17.7 to 44.6 kg/m^2^). There was no relationship between any of the patient baseline characteristics and patients who were included versus excluded based on the availability of KOOS baseline and follow-up scores. Approximately one-third of patients failed to achieve an adequate outcome (ie, did not meet or exceed the MCID) for the KOOS Pain subscale (30%), KOOS Symptoms subscale (21%), KOOS ADL subscale (27%), and KOOS QOL subscale (32%). A slightly higher proportion of patients failed to achieve an optimal outcome (ie, did not meet or exceed the SCB) on the KOOS Pain subscale (33%), KOOS Symptoms subscale (39%), KOOS ADL subscale (27%), and KOOS QOL subscale (36%).

**Table 1:** Demographics and Clinical Characteristics of Study Sample (n = 159)

Item	Overall
	% (n)
Sex	
Male	30.8 (49)
Female	69.2 (110)
Race	
Black	34.0 (54)
White	66.0 (105)
Insurance type	
Private	42.8 (68)
Medicare	32.1 (51)
Medicare advantage	25.2 (40)
Age group, yr	
<65	30.8 (49)
65-74	47.8 (76)
≥75	21.4 (34)
BMI, kg/m^2^	
<25	10.1 (16)
25-29	26.0 (41)
30-34	36.7 (58)
≥35	27.2 (43)
CCI	
0-1	10.1 (16)
2-3	51.6 (82)
>3	38.4 (61)

Item	Overall
	Mean (SD)
Age, yr	67.6 (8.5)
BMI, kg/m^2^	31.7 (5.3)
CCI	3.2 (1.6)
KOOS	
Symptoms	44.7 (19.4)
Pain	40.2 (16.7)
ADL	20.7 (15.7)
QOL	42.9 (18.4)

ADL = activities of daily living, BMI = body mass index, CCI = Charlson Comorbidity Index, KOOS = Knee Injury and Osteoarthritis Outcome Score, QOL = quality of life

### Predictors of Inadequate or Suboptimal Outcome

Tables 2 and 3, respectively, show the predictors of an inadequate and a suboptimal outcome. The strongest predictors of failing to achieve the MCID and SCD for the KOOS Symptoms, KOOS ADL, and KOOS QOL subscale at 6 months were, respectively, the preoperative KOOS Symptoms, KOOS ADL, and KOOS QOL subscale scores. Patients with the best preoperative KOOS Symptoms, KOOS QOL, and KOOS ADL scores (ie, those in the second, third, and fourth quartiles) were notably more likely to have an inadequate or suboptimal outcome at 6 months compared with patients with the worst preoperative scores (first quartile). The preoperative KOOS Pain subscale score was not a statistically significant predictor of achieving the KOOS Pain subscale MCID or SCD. Patient demographics, comorbidities, and BMI were not statistically significant predictors of any outcomes.

**Table 2:** Likelihood of Not Achieving KOOS Minimal Clinically Importance Difference at 6-Month Follow-up After Total Knee Arthroplasty (n = 159)

Item	KOOS Symptoms			KOOS Pain			KOOS ADL			KOOS QOL		
	OR	95% CI	*P*	OR	95% CI	*P*	OR	95% CI	*P*	OR	95% CI	*P*
Race			0.359			0.989			0.958			0.881
White	1.00	Reference		1.00	Reference		1.00	Reference		1.00	Reference	
Black	0.61	0.21-1.76		1.01	0.41-2.46		1.03	0.41-2.59		0.94	0.39-2.23	
Sex			0.315			0.161			0.490			0.893
Male	1.00	Reference		1.00	Reference		1.00	Reference		1.00	Reference	
Female	0.60	0.22-1.63		0.52	0.21-1.30		0.72	0.29-1.81		1.06	0.43-2.64	
Insurance			0.088			0.230			0.201			0.824
Private	1.00	Reference		1.00	Reference		1.00	Reference		1.00	Reference	
Medicare advantage	4.07	1.12-14.8		2.27	0.71-7.21		0.94	0.28-3.13		1.26	0.41-3.84	
Medicare	2.74	0.85-8.79		2.51	0.82-7.62		2.27	0.76-6.76		1.38	0.50-3.84	
Age, yr			0.311			0.972			0.723			0.292
<65	1.00	Reference		1.00	Reference		1.00	Reference		1.00	Reference	
65-75	1.19	0.32-4.46		0.86	0.25-3.02		0.63	0.19-2.11		2.07	0.65-6.59	
>75	0.42	0.07-2.60		0.86	0.18-4.09		0.57	0.12-2.71		1.04	0.22-5.16	
BMI, kg/m^2^			0.417			0.360			0.330			0.124
<25	1.00	Reference		1.00	Reference		1.00	Reference		1.00	Reference	
25-30	0.31	0.07-1.34		1.27	0.29-5.59		0.42	0.11-1.67		0.25	0.06-1.00	
30-35	0.36	0.09-1.46		0.73	0.17-3.21		0.29	0.08-1.10		0.20	0.05-0.75	
>35	0.52	0.12-2.22		1.97	0.44-8.80		0.38	0.09-1.56		0.30	0.07-1.16	
CCI			0.441			0.479			0.215			0.185
1 and 2	1.00	Reference		1.00	Reference		1.00	Reference		1.00	Reference	
2 and 3	0.43	0.08-2.39		0.54	0.12-2.42		0.38	0.09-1.68		0.26	0.06-1.11	
>4	0.28	0.04-2.03		0.35	0.06-2.03		0.72	0.13-4.00		0.30	0.16-1.63	
KOOS			0.029			0.084			0.042			0.014
Quartile 1	1.00	Reference		1.00	Reference		1.00	Reference		1.00	Reference	
Quartile 2	1.23	0.26-5.85		5.13	1.39-18.97		4.99	1.22-20.45		2.17	0.67-7.03	
Quartile 3	2.39	0.56-10.23		4.59	1.13-18.51		3.31	0.77-14.28		2.50	0.82-7.61	
Quartile 4	6.60	1.54-28.30		3.05	0.69-13.40		8.08	1.83-35.74		7.86	2.26-27.34	

ADL = activities of daily living, BMI = body mass index, CCI = Charlson Comorbidity Index, CI = confidence interval, KOOS = Knee Injury and Osteoarthritis Outcome Score, OR = odds ratio, QOL = quality of life

**Table 3:** Likelihood of Not Achieving KOOS Substantial Clinical Benefit at 6-Month Follow-up After Total Knee Arthroplasty (n = 159)

Item	KOOS Symptoms			KOOS Pain			KOOS ADL			KOOS QOL		
	OR	95% CI	*P*	OR	95% CI	*P*	OR	95% CI	*P*	OR	95% CI	*P*
Race			0.739			0.725			0.948			0.837
White	1.00	Reference		1.00	Reference		1.00	Reference		1.00	Reference	
Black	0.86	0.35-2.09		1.16	0.50-2.70		1.03	0.41-2.59		0.92	0.41-2.07	
Sex			0.745			0.143			0.486			0.830
Male	1.00	Reference		1.00	Reference		1.00	Reference		1.00	Reference	
Female	1.16	0.48-2.83		0.52	0.22-1.25		0.72	0.29-1.81		1.10	0.47-2.57	
Insurance			0.179			0.333			0.201			0.861
Private	1.00	Reference		1.00	Reference		1.00	Reference		1.00	Reference	
Medicare Advantage	2.73	0.91-8.19		2.13	0.72-6.27		0.94	0.28-3.13		1.03	0.36-2.99	
Medicare	2.01	0.73-5.55		1.91	0.68-5.36		2.27	0.76-6.76		1.28	0.48-3.42	
Age, yr			0.169			0.778			0.723			0.731
<65	1.00	Reference		1.00	Reference		1.00	Reference		1.00	Reference	
65-75	0.41	0.13-1.26		1.30	0.41-4.10		0.63	0.19-2.11		1.11	0.39-3.16	
>75	0.24	0.05-1.10		0.92	0.21-4.08		0.57	0.12-2.71		0.70	0.16-3.03	
BMI, kg/m^2^			0.424			0.467			0.330			0.328
<25	1.00	Reference		1.00	Reference		1.00	Reference		1.00	Reference	
25-30	0.36	0.09-1.41		0.92	0.23-3.75		0.42	0.11-1.67		0.39	0.10-1.45	
30-35	0.55	0.15-1.99		0.66	0.17-2.64		0.29	0.08-1.10		0.34	0.10-1.21	
>35	0.78	0.21-2.98		1.53	0.38-6.25		0.38	0.09-1.56		0.30	0.08-1.17	
CCI			0.922			0.527			0.215			0.274
1 and 2	1.00	Reference		1.00	Reference		1.00	Reference		1.00	Reference	
2 and 3	0.83	0.18-3.72		0.69	0.17-2.87		0.38	0.09-1.68		0.34	0.09-1.28	
>3	0.72	0.13-3.96		0.43	0.08-2.28		0.72	0.13-3.96		0.36	0.07-1.70	
KOOS			<0.0001			0.123			0.042			0.017
Quartile 1	1.00	Reference		1.00	Reference		1.00	Reference		1.00	Reference	
Quartile 2	5.21	1.31-20.69		4.07	1.27-13.01		4.99	1.22-20.45		1.54	0.53-4.50	
Quartile 3	10.62	2.64-42.81		2.62	0.76-8.98		3.31	0.77-14.28		1.82	0.66-5.04	
Quartile 4	39.67	8.82-178.55		2.18	0.59-8.10		8.08	1.83-35.74		6.05	1.94-18.93	

ADL = activities of daily living, BMI = body mass index, CCI = Charlson Comorbidity Index, CI = confidence interval, KOOS = Knee Injury and Osteoarthritis Outcome Score, OR = odds ratio, QOL = quality of life

**Figure 1:**
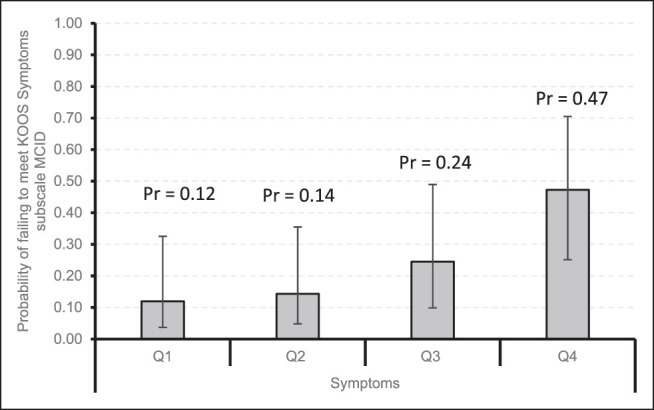
Bar chart showing the probability of failing to meet KOOS Symptoms subscale MCID by quartiles with 95% confidence intervals. KOOS = Knee Injury and Osteoarthritis Outcome Score, MCID = minimal clinically important difference, Pr = probability, Q1 = quartile 1, Q2 = quartile 2, Q3 = quartile 3, Q4 = quartile 4

**Figure 2:**
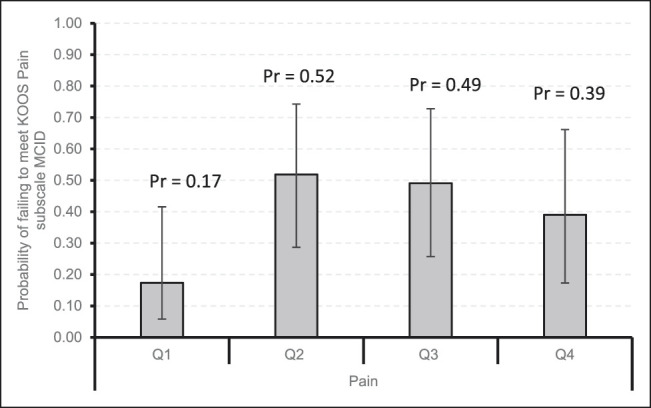
Bar chart showing the probability of failing to meet KOOS Pain subscale MCID by quartiles with 95% confidence intervals. KOOS = Knee Injury and Osteoarthritis Outcome Score, MCID = minimal clinically important difference, Pr = probability, Q1 = quartile 1, Q2 = quartile 2, Q3 = quartile 3, Q4 = quartile 4

**Figure 3:**
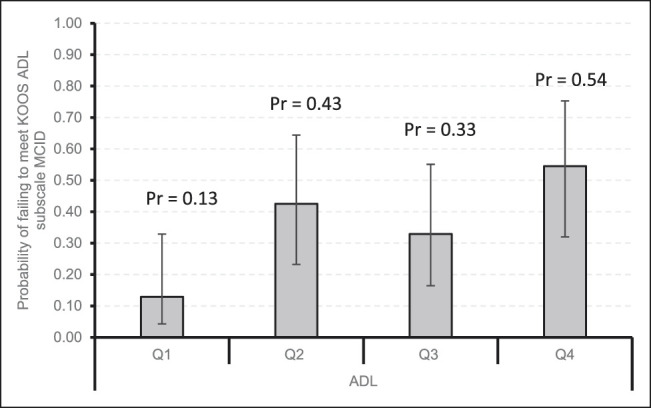
Bar chart showing the probability of failing to meet KOOS ADL subscale MCID by quartiles with 95% confidence intervals. ADL = activities of daily living, KOOS = Knee Injury and Osteoarthritis Outcome Score, MCID = minimal clinically important difference, Pr = probability, Q1 = quartile 1, Q2 = quartile 2, Q3 = quartile 3, Q4 = quartile 4

**Figure 4:**
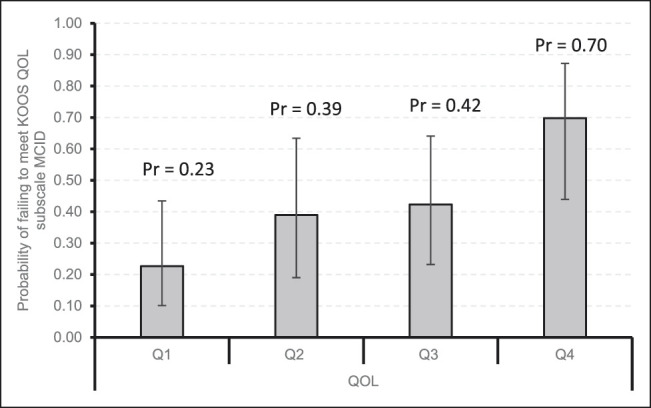
Bar chart showing the probability of failing to meet KOOS QOL subscale MCID by quartiles with 95% confidence intervals. KOOS = Knee Injury and Osteoarthritis Outcome Score, MCID = minimal clinically important difference, Pr = probability, Q1 = quartile 1, Q2 = quartile 2, Q3 = quartile 3, Q4 = quartile 4, QOL = quality of life

Figures 1–4 demonstrate that patients with higher baseline KOOS subscale scores were more likely to not meet the MCID. Notably, patients with the best preoperative KOOS scores (fourth quartile) were 7 to 8 times more likely to have an inadequate outcome on the KOOS Symptoms (odds ratio [OR] 6.6, 95% confidence interval [CI] 1.54 to 28.30), KOOS ADL (OR 8.08, 95% CI 1.83 to 35.74), and KOOS QOL (OR 7.86, 95% CI 2.26 to 27.34) subscales.

## Discussion

In this study, approximately 30% of patients failed to achieve an adequate outcome 6 months after TKA. The failure rate found in the present study is slightly higher than that reported in other studies (about 20% of TKA patients report dissatisfaction with surgery results^17^) and substantially higher than the 9% to 15% failure rate reported by Lyman et al.^8^ We believe the higher failure rate in the present study is reflective of an especially ill, debilitated, and socially disadvantaged patient cohort. Although 67% of patients in the study by Lyman et al had a CCI score of 0 (no major comorbidities), only 10% of our study cohort had a CCI score of 0 or 1. The mean preoperative KOOS ADL score in the study by Lyman et al was 55 compared with 21 in our study. Although a worse (lower) KOOS ADL score may allow for more improvement, the degree of improvement after TKA may be more limited in patients with especially low physical function who have notable medical comorbidities. Finally, although Lyman et al did not report the racial composition of their sample, the racial composition of total hip arthroplasty patients treated at their study site (Hospital for Special Surgery, New York, NY) is approximately 97% white and 3% black^18^ compared with 66% white and 34% black patients in our sample.

Patients with better preoperative knee-related symptoms, function, and QOL scores were most likely to have an inadequate or suboptimal outcome at 6 months after TKA. This is not surprising because patients with better preoperative pain and function tend to have less room to improve than those with more severe knee-related symptoms.^11^ These findings are consistent with the literature, which has found preoperative pain and function to be the most consistent predictors of change in PROs after TKA.^9,10^ It is important to note that all eligible patients may benefit from TKA and should not be denied surgery based on preoperative characteristics alone.^11^ Instead, clinicians should focus on how to improve outcomes in patients at greatest risk for an inadequate outcome, perhaps by setting clear expectations for the amount of improvement to be expected before surgery.

Worse preoperative pain has been consistently associated with PROs after TKA.^5,9,10,11,12,13,14^ Our data showed a nonsignificant trend for less preoperative pain predicting failing to achieve an adequate outcome on the KOOS Pain subscale, but other preoperative KOOS subscales were much more significantly and consistently predictive of outcomes. A larger sample size may have been required to demonstrate a statistically significant relationship between preoperative KOOS pain and study outcomes. We also found that no demographic or clinical variables (aside from preoperative PROs) were statistically significant independent predictors of TKA outcomes. Although some studies have shown that specific demographic characteristics are strongly associated with TKA outcomes, findings across studies are often contradictory, and the strength of associations between predictors and outcomes is unclear because of considerable variability across studies in sample sizes, outcome measures, and analytic methods.^9,10^ We hope to see additional studies that use the KOOS MCID and SCB values as outcomes so that findings can be directly compared with the results of this study.

In addition to the inherent limitations of retrospective studies, this study was also limited by a small sample size, treatment by a single surgeon, restricted types of variables available for inclusion in the analyses, and a relatively short follow-up period. The relatively small sample size may have resulted in inadequate power to detect a statistically significant relationship between KOOS Pain and outcomes. All patients were treated by a single surgeon in the New Orleans greater metropolitan area, which limits the generalizability of the results but reduces the effect of variable surgical practices and surgeon skill on patient outcomes. Some studies have shown that psychosocial variables, including patient expectations,^19–21^ preoperative mental health,^22^ and socioeconomic status,^10^ are associated with TKA outcomes; however, these potentially important variables were not available for inclusion in the analysis. In particular, patient expectations before surgery are consistently associated with patient satisfaction after TKA.^19,20^ This stresses the importance of understanding patient expectations preoperatively and setting realistic expectations for postsurgical outcomes, especially for patients who are less likely to experience large gains after surgery.^19–21^ Our measure of comorbidities, the CCI, was developed to predict mortality and may not be the best measure for assessing the effect of comorbid conditions on outcomes in patients with osteoarthritis.^23,24^ We were only able to assess outcomes up to 6 months after TKA. Although prediction of longer term outcomes may be desirable, the most notable progress after TKA takes place during the first 6 months after surgery.^25,26^

This study is the first to identify patient characteristics that are associated with inadequate and suboptimal outcomes per the KOOS subscale thresholds validated in a recent study. Approximately one-third of patients who underwent TKA in this study failed to achieve an adequate outcome, a success rate somewhat lower than that has been generally reported in the literature but can be explained by the study's particularly ill, debilitated, and socially disadvantaged patient cohort. Consistent with previous studies, we found that patients who had the least severe knee symptoms before surgery were 7 to 8 times more likely to fail to achieve an adequate outcome compared with patients with the most severe symptoms. Given the high risk for dissatisfaction in patients with comparably less severe knee problems before surgery, surgeons are advised to counsel these patients about the degree to which they can expect improvement after TKA.
